# Treatment of Heparin-Induced Thrombocytopenia (HIT) With Fondaparinux in a Patient With Liver Dysfunction

**DOI:** 10.7759/cureus.88304

**Published:** 2025-07-19

**Authors:** Spiros Delis, Dimosthenis Chrysikos, Dimitrios Schizas, Eugenia Charitaki, Nikolaos Taprantzis, Vasiliki Kylafa, Amir Shihada, Theodore Troupis

**Affiliations:** 1 Department of Surgery, Konstantopoulio General Hospital, Nea Ionia, GRC; 2 Department of Anatomy, National and Kapodistrian University of Athens School of Medicine, Athens, GRC; 3 First Department of Surgery, Laiko General Hospital of Athens, Athens, GRC; 4 Department of General Surgery, Konstantopoulio General Hospital, Nea Ionia, GRC

**Keywords:** fondaparinux, hepatectomy, hit, thrombocytopenia, type ii heparin

## Abstract

Type II heparin-induced thrombocytopenia (HIT) is an immune-mediated reaction that usually develops 5-10 days after heparin administration and is mediated by antibodies against a neoantigen of heparin and platelet factor 4 complex. The condition is strongly associated with thrombosis, such as pulmonary embolism. Diagnosis of HIT in patients who underwent liver resection is a challenge compared to other surgical patients.

We present the case of a chronic carrier of hepatitis B who presented with a single hepatocellular carcinoma (HCC) in the right lobe. After undergoing right lobectomy without blood transfusion, he was provisioned with low-molecular-weight heparin (LMWH), which was tolerated well. However, after a sudden decrease in platelet count and the onset of tachycardia and shortness of breath, the patient was found to have a partially occluded right pulmonary artery due to pulmonary embolism. After initiating lepirudin, it was discontinued due to unwanted side effects. Thus, fondaparinux was administered, which proved to be effective in increasing the platelet number and resolving the pulmonary embolus. A daily monitoring of anti-factor Xa time was applied using RECALMIX (Amax-Accuclott Heptest, Trinity Biotech, Jamestown, New York, United States). During the first week of fondaparinux administration, initial platelet count values began returning to normal values with resolution of the pulmonary embolus. The patient was discharged in stable condition.

This is the first report that shows effective maintenance anticoagulation with fondaparinux of a patient with cirrhosis and post-hepatectomy HIT syndrome complicated by pulmonary embolism. Further studies are required to establish fondaparinux as a new alternative anticoagulant in surgical patients with HIT.

## Introduction

Thrombocytopenia is a serious side effect following the administration of heparin or low-molecular-weight heparin (LMWH). Type II heparin-induced thrombocytopenia (HIT) is an immunological medical condition that typically emerges 5-10 days following heparin exposure. This reaction is a result of antibodies targeting a newly formed antigen, composed of heparin and platelet factor 4, which makes up the Hp-PF4 complex. The condition is strongly associated with thrombosis, such as symptomatic venous thromboembolism or pulmonary embolism [1].

Although HIT syndrome can be suspected in surgical patients after progressive thrombocytopenia and heparin administration, diagnosis is less straightforward in patients who have undergone liver resection. The calculation of the 4T score is widely used as a means to assess the probability of HIT. The factors that determine the final score are based on thrombocytopenia, timing of platelet count fall, thrombosis, and other non-evident causes [2]. In cases of high probability, an additional heparin-PF4 IgG enzyme-linked immunosorbent assay (ELISA) test should be performed in order to determine the exact likelihood of HIT presence. Moreover, other conditions often associated with hepatectomy in cirrhotic liver, such as sepsis, disseminated intravascular coagulation (DIC), or blood loss, followed by a hematoma formation, especially in cirrhotic livers, should be ruled out [3].

The risk of acute blood loss should be taken into consideration when forming an anticoagulation therapy in patients who underwent liver resection complicated by HIT, while the use of danaparoid may prove to be useful through its cross-reaction with HIT antibodies [4,5]. A direct thrombin inhibitor, such as r-Hirudin, in combination with a coumarin-class anti-vitamin K agent is the preferred treatment. A coumarin-class anticoagulant in acute HIT can be deleterious by predisposing to micro-thrombosis via protein C depletion, already present due to decreased synthesis from a marginal remnant liver parenchyma [6]. Fondaparinux, a synthetic pentasaccharide, is commonly employed in the early management of pulmonary embolism related to HIT, thanks to its ability to selectively inhibit factor X [7]. Yet, evidence on its safety and efficacy in cirrhotic patients undergoing major liver surgery remains limited.

To our knowledge, this is the first documented case demonstrating successful prolonged anticoagulation management using fondaparinux in a patient with cirrhosis and post-hepatectomy HIT syndrome complicated by pulmonary embolism. This case highlights a potentially valuable treatment strategy in a uniquely high-risk population, where conventional anticoagulation options may be limited or contraindicated. A review of the literature is also reported.

## Case presentation

**Figure 1:**
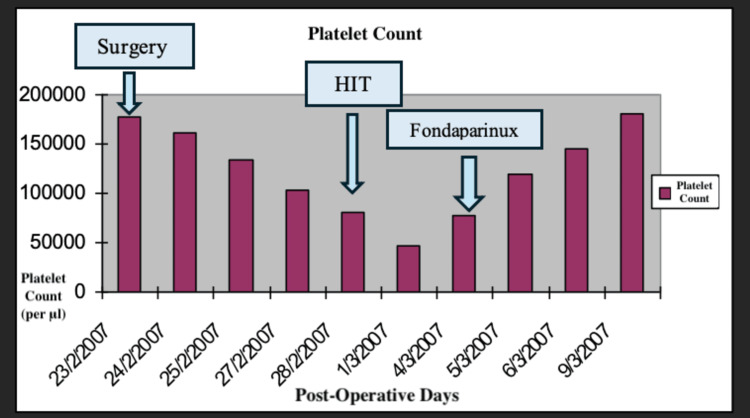
Platelet count throughout the postoperative days HIT: heparin-induced thrombocytopenia

A 75-year-old Caucasian man (weighing 80 kg), a chronic carrier of hepatitis B, presented with a single hepatocellular carcinoma (HCC) in the right liver lobe. The patient had a normal platelet count and Child A cirrhosis with a Model for End-Stage Liver Disease (MELD) score of 6. He underwent right lobectomy without blood transfusion, remained overnight in the intensive care unit (ICU), and was transferred in good condition to the floor the post-op day (POD) 1. He received a daily prophylactic dose of LMWH (Clexane 40 mg s.c./day) for a number of days and tolerated the procedure well with full mobilization since POD 2. However, during the sixth POD, his platelet count started falling progressively to reach 45000/μl (Figure 1). 

**Figure 2:**
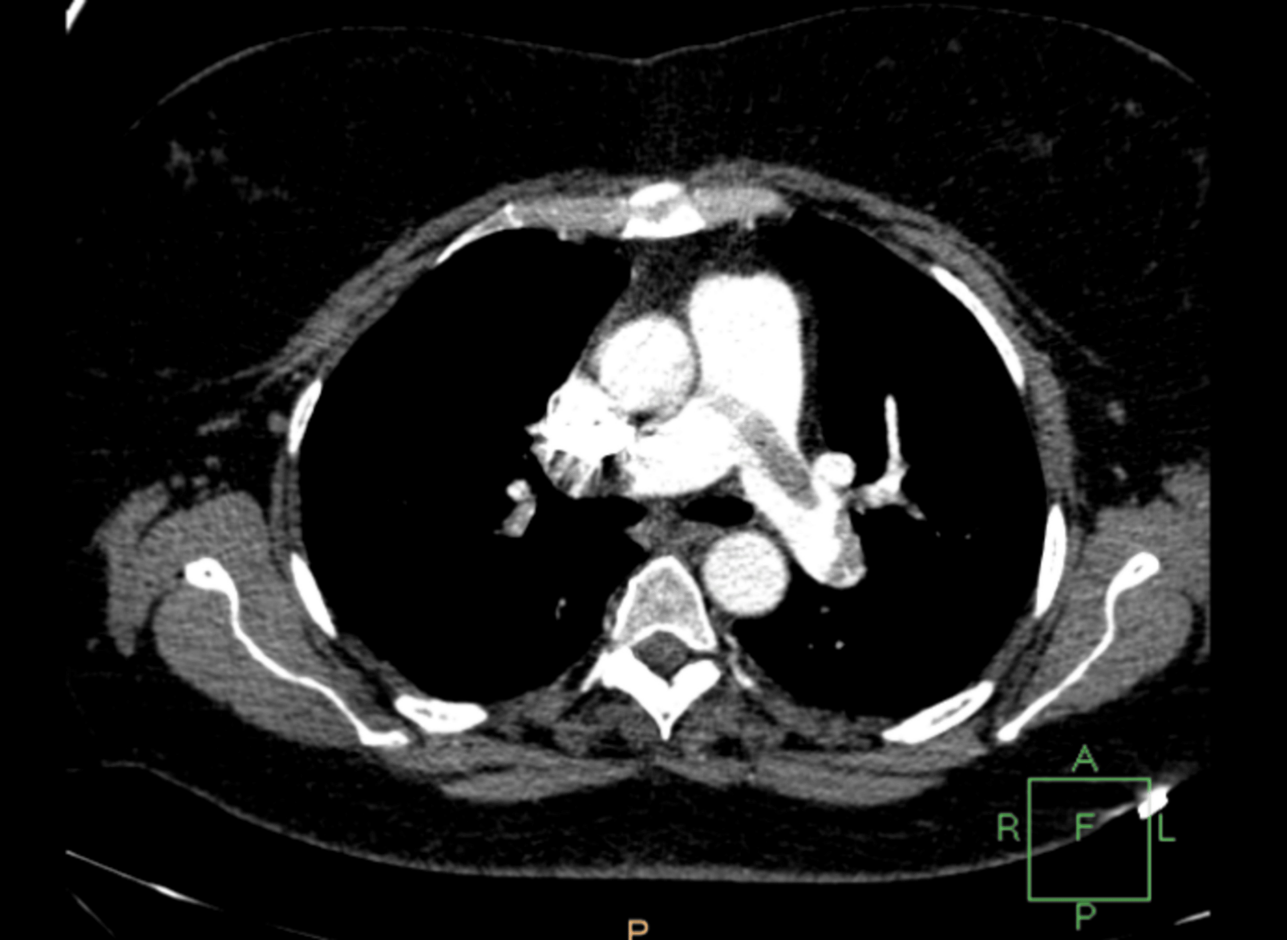
Computed tomography angiography showing pulmonary embolism

**Figure 3:**
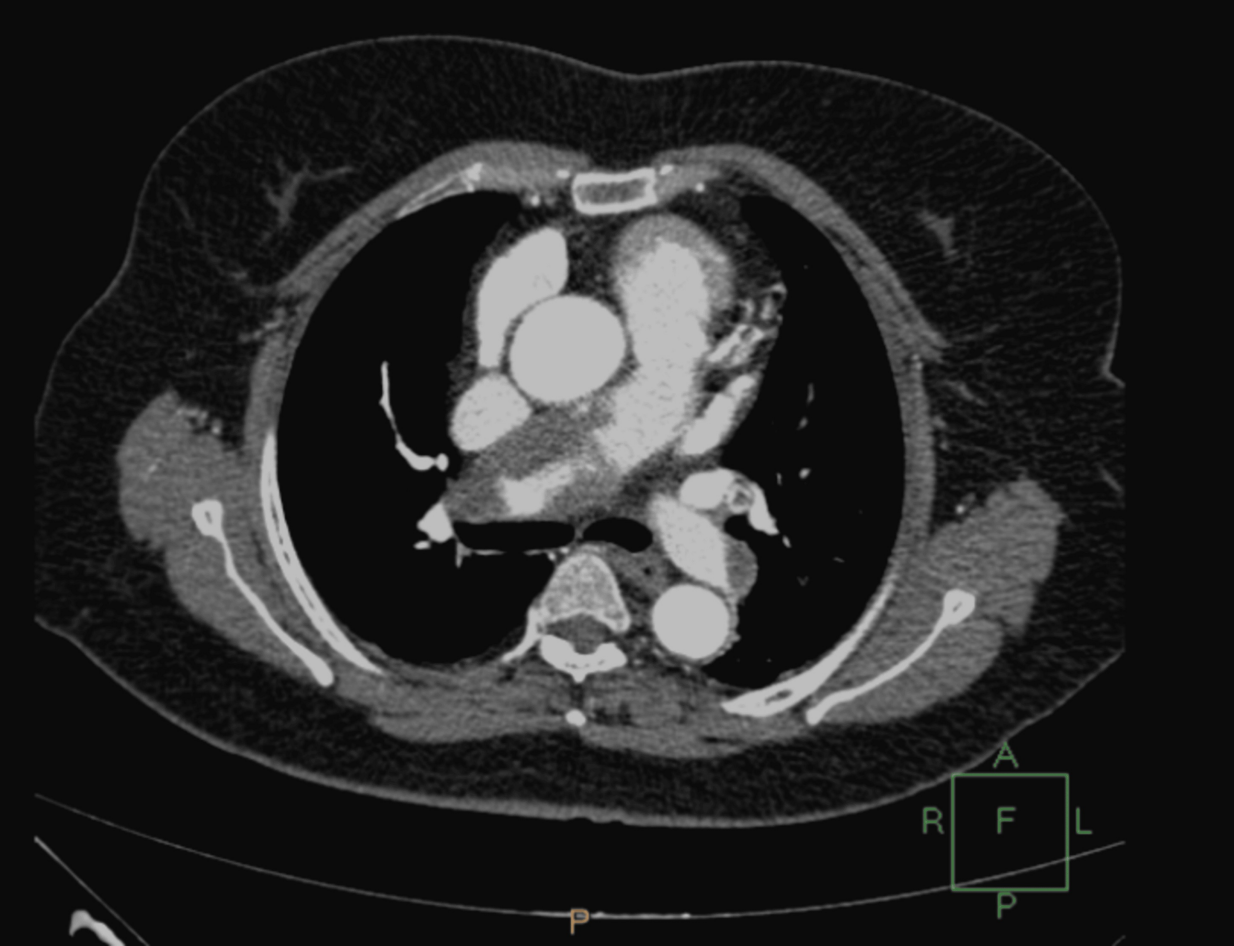
Computed tomography angiography showing pulmonary embolism

**Figure 4:**
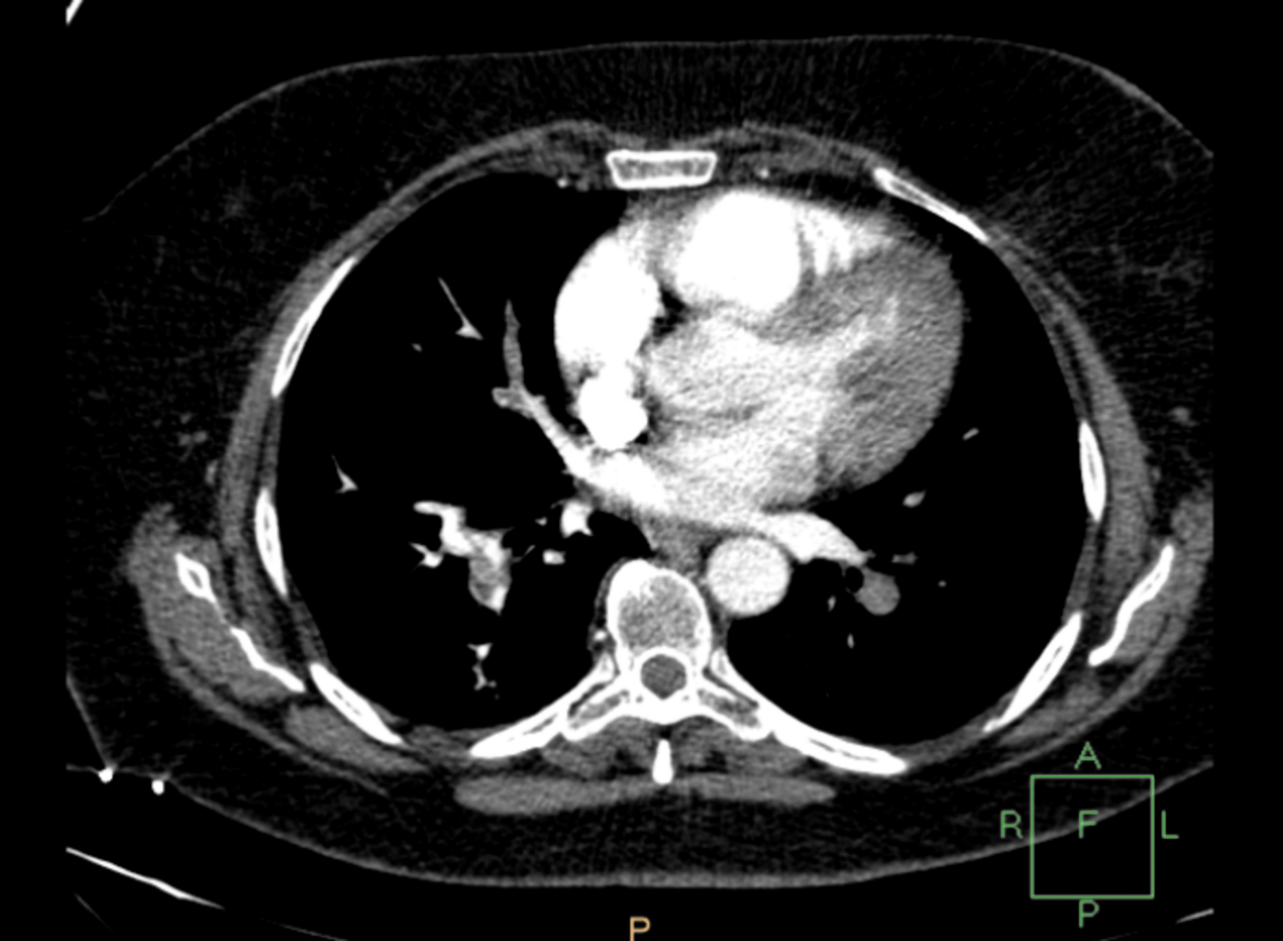
Computed tomography angiography showing pulmonary embolism

**Figure 5:**
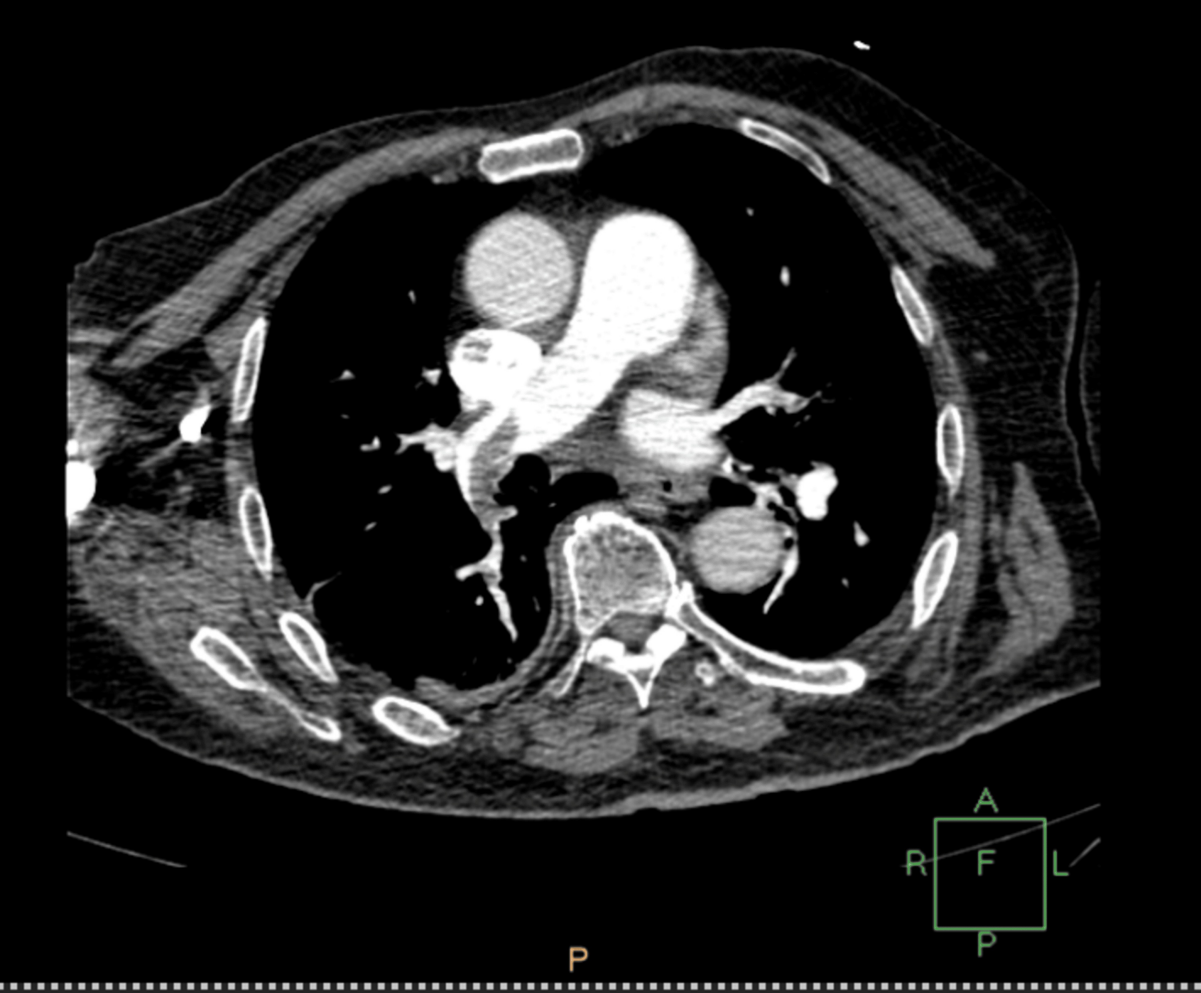
Computed tomography angiography showing pulmonary embolism

The patient complained of shortness of breath and tachycardia with subsequent respiratory alkalosis and low PO2 (60 mm Hg). Although chest X-ray didn't reveal any abnormal findings, computed tomography (CT) angiography revealed a partially occluded right pulmonary artery due to pulmonary embolism (Figures 2-5).

Doppler ultrasonography scan of the lower extremities didn't show deep vein thrombosis. Bleeding, sepsis, and DIC were ruled out. Sepsis was excluded because of the patient's non-febrile state. White blood count was in normal range (8.61×10^3^/μl with the number of neutrophils 5.56×10^3^/μl), and cultures of urine and blood samples were negative for bacterial infection, although they were tested three times. DIC was also excluded because of the absence of bleeding signs without significant prolongation of prothrombin time (PT) and activated partial thromboplastin time (APTT). Fibrinogen was in normal range, and a blood smear didn't reveal schistocytes. Liver enzyme values were in normal range, although poor synthetic liver function by means of antithrombin, protein C, and factor VII was appreciated. The level of antithrombin III in plasma was 31% (activity), of protein C was 24% (activity), of factor VII was 36% (activity), and of factor V was 65% (activity). Daily monitoring of protein C and factor VII was pursued as part of a broader coagulation panel to evaluate liver synthetic function and procoagulant/anticoagulant balance in the patient to exclude DIC and fluctuating coagulopathy. While this is not standard per guidelines, it was used to guide transfusion strategy and monitor the risk of thrombosis versus bleeding.

**Figure 6:**
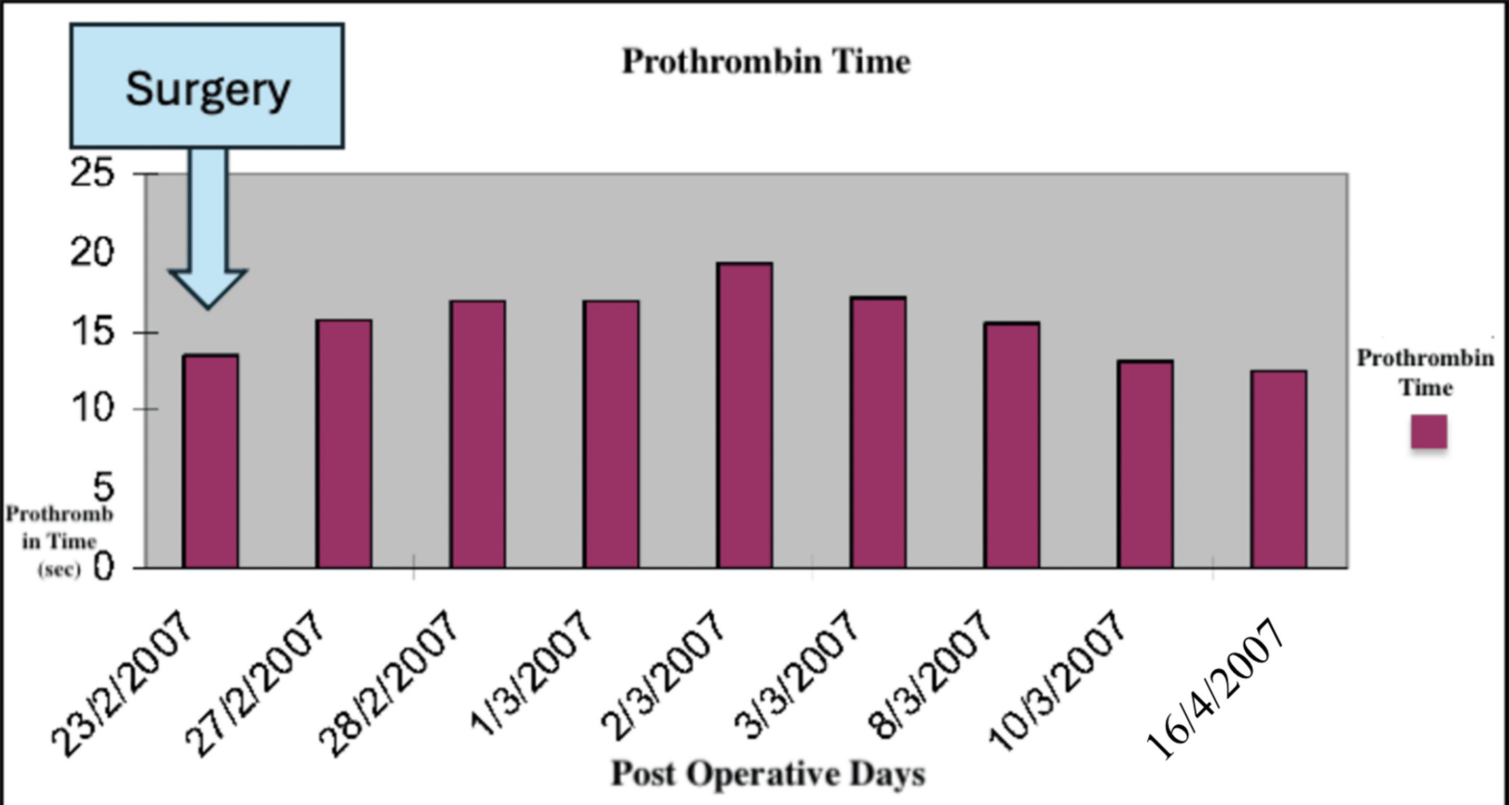
Prothrombin time throughout the postoperative days

**Figure 7:**
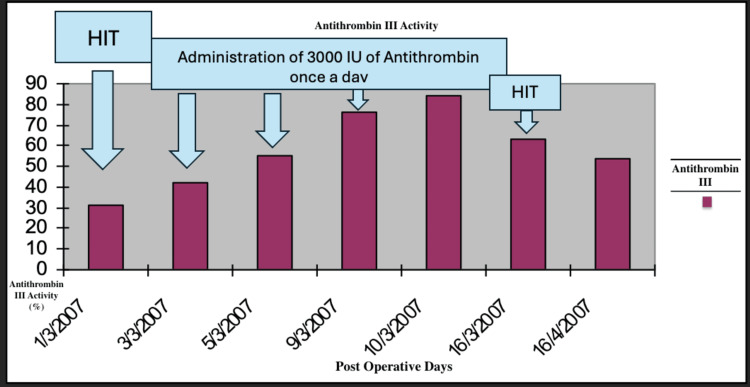
Antithrombin III activity throughout the postoperative days HIT: heparin-induced thrombocytopenia

**Figure 8:**
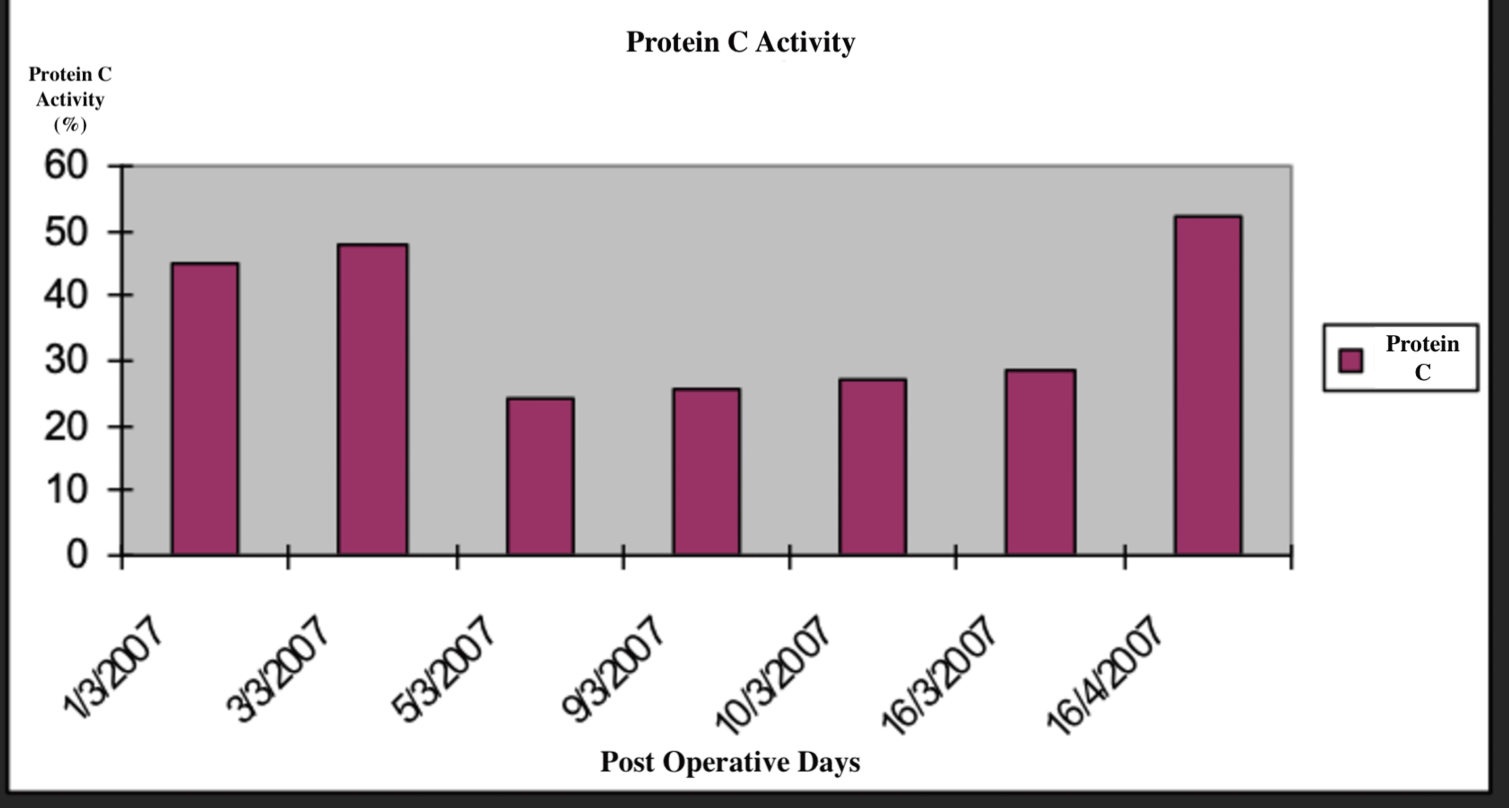
Protein C activity throughout the postoperative days HIT: heparin-induced thrombocytopenia

**Figure 9:**
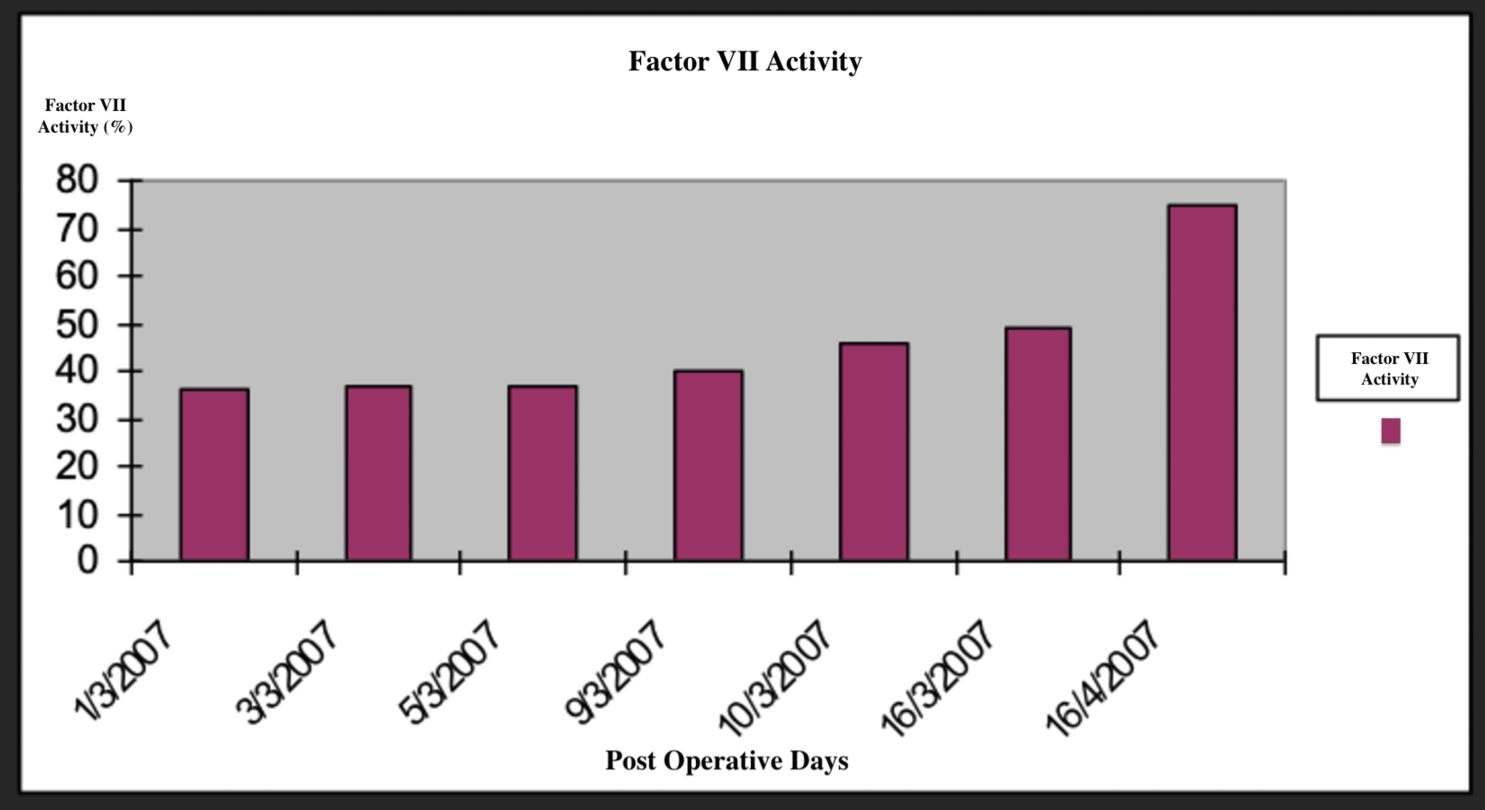
Factor VII activity throughout the postoperative days

A 4T score was calculated on the day of presentation and was 6 (2 points for thrombocytopenia, 2 for timing, 1 for thrombosis, and 1 for other causes not evident). Given this high pretest probability, a heparin-PF4 IgG ELISA was ordered. The test returned positive with an optical density (OD) of 2.4, which is above the threshold typically considered highly suggestive of HIT. Overall, the combination of a high 4T score (>5) and ELISA OD >2 yields a >95% probability of HIT. Α confirmatory serotonin release assay (SRA) was performed, which depicted a serotonin release of more than 50%. The SRA was performed using both low and high heparin concentrations. The assay showed activation with low-dose heparin and inhibition with high-dose heparin, which is consistent with a positive result for HIT. Lepirudin was initiated as an alternative anticoagulation agent, and its effectiveness was measured every day using the values of the APTT. Two days after the initiation of lepirudin administration, bleeding was noted from the Jackson-Pratt drain and the urinary catheter. At the time of bleeding, the patient's APTT was 110 seconds, exceeding the therapeutic target (1.5-2.5× baseline), thus indicating a supratherapeutic anticoagulation level. This was treated effectively by the interruption of the medication and fresh frozen plasma administration. Lepirudin was replaced by fondaparinux 7.5 mg s.c. for a three-month period. This decision was made due to the local unavailability of bivalirudin and argatroban at that time. Although direct oral anticoagulants (DOACs) (e.g., apixaban and rivaroxaban) are recognized alternatives per American Society of Hematology (ASH) guidelines [8], they were not selected due to the active bleeding, necessitating an agent with parenteral administration and shorter half-life, and the patient's unpredictable enteral absorption in critical illness. A daily monitoring of anti-factor Xa time was applied using RECALMIX (Amax-Accuclott Heptest, Trinity Biotech, Jamestown, New York, United States). During the first week of fondaparinux administration, initial platelet count values began returning to normal values with resolution of the pulmonary embolus, which was confirmed via Doppler ultrasound and CT pulmonary angiogram. The patient was discharged in stable condition (Figures 6-9). Table 1 presents the laboratory findings of the patient. 

**Table 1:** Patient laboratory findings SRA: serotonin release assay

Test	Patient result	Reference range	Units
PO2 levels	60	80-100	mm Hg
White blood count	8.61x10^3^	4500-11000	/μl
Neutrophil count	5.56x10^3^	2500-7000	/μl
Serotonin release increase (by SRA)	50%	<20% (negative SRA)	-
Anti-PF4/heparin antibodies	Positive (+)	-	-
Urine sample culture	Negative (-)	-	-
Blood sample culture	Negative (-)	-	-

## Discussion

Type II HIT is an immune-mediated reaction by means of the activation of IgG class platelet antibodies that recognize complexes of platelet factor 4 and heparin. It is strongly associated with thrombosis by causing platelet, endothelium, and monocyte activation. The clinical profile of HIT includes the 4T clinical score popularized by Warkentin and associates [9,10]. Thrombocytopenia with thrombosis 5-14 days after the initiation of heparin administration, in the absence of other explanations, establishes a clinical diagnosis of HIT, which is further confirmed by serologic tests such as SRA, enzyme immunoassay (EIA), and heparin-induced platelet aggregation (HIPA) test [1]. In our case, in accordance with the ASH guidelines [8], a high clinical 4T score (=6) and an ELISA OD>2 were needed to prove the diagnosis. Thrombocytopenia is defined as a decrease in platelet count of 50% or more from the postoperative peak, rather than being based on an absolute platelet count threshold. Factors affecting the frequency of HIT include the type of heparin (unfractionated heparin >LMWH), the type of patient (surgical>medical), and gender (female>male) [1,11]. The clinical picture involves thrombosis of lower limb arteries, deep veins of the lower extremities, and pulmonary arteries, although skin lesions at heparin injection sites, acute systemic reactions, and adrenal necrosis are relatively specific to the syndrome [1,12].

Despite the fact that the detection of pathogenic HIT antibodies is possible through various sensitive assays, restrictions and limitations still exist. Currently, besides EIAs that measure antibodies to heparin-platelet factor 4 complex 3, functional assays are used in vitro: the SRA test, the HIPA test, and the platelet aggregation test. The limitations include delays in access to testing and the detection of non-pathogenic antibodies, which can lead to diagnostic uncertainty [13]. Assuming that a single positive test is able to confirm the presence of HIT, the risk for overdiagnosis increases significantly, especially in patients with a low pretest probability. To minimize this risk, serologic testing should generally be prioritized and preserved for individuals with an intermediate or high 4T score. Apart from this process, the usage of IgG-specified lateral flow immunoassay (LFIA) has shown really positive results [14].

Diagnosis of HIT is more challenging in patients who have undergone liver resection due to multiple causes of thrombocytopenia postoperatively. Portal hypertension, hematoma, sepsis, and medications are associated with thrombocytopenia after liver surgery and have to be excluded from the differential diagnosis of HIT [15]. Furthermore, DIC is a possible sequela, making the diagnosis of HIT more challenging. However, a diagnosis of HIT can be supported by a high 4T clinical score, in combination with the presence of vascular occlusions during the administration of prophylactic anticoagulation and a substantial decrease in platelet count, accompanied by positive anti-PF4/heparin antibodies by EIA [16]. In our case of HIT after right lobectomy, the patient developed progressive thrombocytopenia and right pulmonary embolism with positive serology for HIT, as demonstrated by the graphical figures and confirmed by CT angiography images. D-dimers were increased with concomitant but not significant prothrombin prolongation and low antithrombin and protein C levels. The liver enzymes were normal, and the patient remained non-febrile with good postoperative recovery. Sepsis, hematoma, and DIC were excluded due to the negative laboratory findings, in addition to the absence of other measurements, such as significant prolongation of PT and APTT.

The liver is the site of production of all coagulation factors, except von Willebrand factor and factor VIII [17]. Liver dysfunction results in deficiencies of all the coagulation factors and the inhibitors of coagulation. However, some of these changes, such as prolongation of PT, reduced fibrinogen, and D-dimer increase, are commonly presented in other conditions (DIC). In this regard, it is mandatory to differentiate liver dysfunction from DIC, especially after liver surgery and background cirrhosis [18]. The laboratory tests which are frequently abnormal in DIC include the following: thrombocytopenia, increased D-dimer value, PT prolongation (due to the reduction of clotting factors, especially factor V), reduced fibrinogen level, and identification of fragmented red cells in peripheral blood smear (formed after injury by fibrin strands, endothelial cell damage, or tumor cell emboli). This differentiation becomes more complex in the rare case of HIT syndrome and marginal liver function [19-22]. In our case report, platelet count reached 45000/μl after the administration of LMWH with subsequent thrombotic sequela. PT was slightly prolonged, APTT was normal, and fibrinogen (as an acute-phase reactant) was increased postoperatively. 

Due to the presence of HIT antibodies, discontinuation of LMWH and alternative anticoagulation is required, such as lepirudin. Lepirudin inhibits thrombin directly although the risk of bleeding remains high and current trends are to avoid the initial lepirudin bolus with close monitoring of APTT at four-hour intervals [1,23]. Despite close monitoring, our patient developed bleeding from the drain, and hematuria responded to fresh frozen plasma administration and the discontinuation of lepirudin. Due to protein C and antithrombin depletion in our case, coumarin anticoagulation was contraindicated to avoid further thrombotic complications. This decision was also supported by ASH guidelines [8], which state that coumadin anticoagulation should not be initiated until the platelet count exceeds 150×10⁹/L. Our sense is that protein C, antithrombin, and factor VII depletion were associated with poor synthetic capability from the cirrhotic liver remnant, although liver dysfunctions, such as jaundice and ascites, were not documented. Therefore, we started treatment with the provision of fondaparinux, which exerts its anticoagulant effect through the targeted inhibition of activated factor X. Unlike heparin, fondaparinux does not bind to PF4, preventing the formation of immune complexes responsible for type II HIT [24-27]. Recent clinical trials have also shown encouraging outcomes, demonstrating fondaparinux to be a successful and safe option for the primary therapeutic approach for pulmonary embolism [28]. Although in the literature [29] a subcutaneous administration of 2.5 mg is reported for both prophylaxis and treatment of thromboembolism, there is uncertainty regarding optimal dosing. Thus, we chose a higher dose of 7.5 mg subcutaneous injection daily for three months and a simultaneous administration of antithrombin concentrate at a cumulative dose of 103,000 units over seven days to correct the documented low antithrombin III activity (<50%), which was believed to contribute to inadequate anticoagulant response and the progression of pulmonary embolism. Therapy was continued until the normalization of antithrombin III levels and radiological resolution of the pulmonary embolism. Supportive correction of antithrombin III activity was performed during the first week due to low measured levels. Purified factor Xa was used to determine the anticoagulant effects of fondaparinux. Using RECALMIX, the anti-factor Xa time was monitored daily and remained in the therapeutic range. Platelet levels normalized and were maintained steadily in the weeks that followed.

As far as we are aware, this report includes the first described clinical case that utilizes fondaparinux as a successful anticoagulation management for a patient who has undergone liver resection. In more than 200 cases of liver resection in our Liver Surgical Unit, we defined one case of HIT complicated by thrombosis (rate 0.5%). Other studies in patients with liver metastases from colorectal cancer report an incidence rate, regarding deep vein thrombosis, of about 2.1%, using the same preventive measures preoperatively and postoperatively [30]. Although the true incidence of positive HIT antibodies is not known after liver resection, the only report in the literature is related to liver transplantation. The percentage of HIT antibody-positive patients in a recent cohort of 52 living related liver recipients was 0.5% preoperatively, 5.6% on POD 7, and 5.6% on POD 14. The authors concluded that the occurrence of HIT after liver transplantation was uncommon [31]. Given that fondaparinux is the longest-acting anticoagulant and is associated with a high risk of thrombocytopenia as well as postoperative bleeding, this outcome is particularly noteworthy. Safety regulations and key contradictions should be applied for patients who present with bleeding disorders, renal disorders, or hypersensitivity to fondaparinux [32]. While this case highlights a successful outcome, it is important to acknowledge the inherent limitations of a single-patient case report, along with the absence of comparative evaluation with other possible treatment options for HIT, such as DOACs, which restricts generalizability. Nevertheless, it offers valuable clinical insight into the use of fondaparinux for this specific clinical context. It should be noted that additional studies and research need to be conducted, so as to acquire enough data regarding the role and effectiveness of fondaparinux in these kinds of patients. 

## Conclusions

HIT syndrome complicated by thrombosis in patients who underwent liver dissection is rare and difficult to diagnose. This report presents a unique approach and management method for an interesting and complicated clinical case. Fondaparinux can be a new alternative anticoagulant in hepatobiliary surgical patients with HIT, but further studies are required to establish this proposition.
